# Antithrombin inhibition using nanobodies to correct bleeding in hemophilia

**DOI:** 10.15252/emmm.202012143

**Published:** 2020-03-25

**Authors:** Jamie M O'Sullivan, James S O'Donnell

**Affiliations:** ^1^ Irish Centre for Vascular Biology Royal College of Surgeons in Ireland Dublin 2 Ireland

**Keywords:** Haematology, Immunology

## Abstract

In this issue of *EMBO Molecular Medicine*, Barbon *et al* describe a new approach to rebalancing coagulation in patients with hemophilia (PWH) through targeted inhibition of anticoagulant antithrombin (AT) (Barbon *et al*, 2020). In contrast to previous studies that used RNA interference (RNAi) therapy to reduce AT levels (Sehgal *et al*, 2015; Pasi *et al*, 2017), the authors utilized llama‐derived single‐domain antibodies (sdAbs or nanobodies) to inhibit AT activity (Fig 1). These engineered sdAbs successfully restored thrombin generation in hemophilic plasma and corrected bleeding phenotype in a murine hemophilia model. Furthermore, long‐term AAV8‐mediated hepatic expression of the sdAb was well tolerated and associated with a sustained correction in bleeding in hemophilia A and B mice. Collectively, these exciting data uncover a novel AT‐targeting approach that may be useful as an alternative therapy for restoring normal hemostasis in PWH.

## Graphical abstract

J. O'Donnell and J. O'Sullivan discuss the challenges of rebalancing coagulation in patients with hemophilia, and the latest advances based on single‐domain antibodies targeting antithrombin from P. Lenting and colleagues (in this issue of *EMBO Molecular Medicine*).

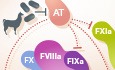

Hemophilia A and B are X‐linked inherited bleeding disorders that result from deficiencies in procoagulant factor VIII (FVIII) or factor IX (FIX), respectively (Mannucci, 2002). Hemophilia is further classified as mild, moderate, or severe on the basis of residual plasma clotting factor levels. There are an estimated 400,000 PWH worldwide, and approximately half of these patients have severe hemophilia. Patients with severe hemophilia have plasma FVIII or FIX levels < 0.01 IU/ml (< 1% normal) and typically develop spontaneous joint and muscle hematomas (Mannucci, 2002). Recurrent joint bleeds commencing in early childhood result in progressive musculoskeletal deterioration and irreversible hemophilic arthropathy. In addition, PWH also experience increased bleeding following trauma or surgery. To reduce bleeding episodes, PWH require lifelong treatment with FVIII or FIX replacement. Previous studies have shown that regular FVIII/FIX administration reduces the number of spontaneous joint bleeds and significantly improves quality of life (Manco‐Johnson *et al*, 2007). Nevertheless, there are important limitations with respect to standard hemophilia prophylaxis treatment. First, since the plasma half‐lives of both FVIII and FIX are short, prophylaxis typically requires intravenous infusions to be administered 2–3 times each week. Second, the cost implications in delivering prophylaxis are significant (estimated $200,000 per annum in USA) and inevitably result in limited access to prophylaxis particularly in developing countries (Thorat *et al*, 2018). Finally, up to 30% of patients with severe hemophilia A and 5% of patients with severe hemophilia B develop neutralizing anti‐FVIII or anti‐FIX antibodies following prophylaxis that render further replacement therapy ineffective.

In an effort to improve treatment options for PWH, a number of alternative therapeutic approaches have been explored in recent years (Callaghan *et al*, 2018). Emicizumab (Hemlibra; Chugai Roche) is a humanized bispecific antibody administered subcutaneously that has a half‐life of 3–4 weeks (Nogami & Shima, 2019). By binding to both FIXa and FX, emicizumab mimics the normal procoagulant cofactor role of FVIIIa. Recent phase 3 clinical trials have demonstrated the clinical efficacy of emicizumab in patients with hemophilia A (Nogami & Shima, 2019). Consequently, emicizumab has recently been licensed in a number of different jurisdictions for prevention of bleeding episodes in patients with severe hemophilia A with or without FVIII inhibitors. Although generally well tolerated, some cases of venous and arterial thrombotic complications have been described in PWH receiving emicizumab (Nogami & Shima, 2019).

Physiological hemostasis involves a tightly regulated balance between procoagulant factors on the one hand, opposed by a series of counter‐regulatory anticoagulant pathways on the other (O'Donnell *et al*, 2019). Consequently, an alternative approach to restoring hemostatic balance in PWH is through targeted inhibition of specific anticoagulant pathways. Antithrombin (AT) is plasma serine protease inhibitor (serpin) that regulates the procoagulant activity of a number of activated clotting factors, including thrombin and activated factor X (FXa; Preston *et al*, 2019; Fig 1). Patients with inherited antithrombin deficiency have significantly increased risk of venous thromboembolism (VTE). Recent studies have reported that targeted antithrombin inhibition using RNAi therapy (Fitusiran; Alnylam Pharmaceuticals) can induce dose‐dependent reductions in plasma AT levels. Once‐monthly fitusiran therapy administered by subcutaneous injection was sufficient to markedly reduce plasma AT levels (< 30% of baseline levels) resulting in significantly enhanced thrombin generation in hemophilic patients with either FVIII or FIX deficiency (Sehgal *et al*, 2015; Pasi *et al*, 2017). Preliminary data suggest that this RNAi treatment, which is now in late‐stage clinical trials, was also associated with a reduction in annualized bleeding rates in PWH (Pasi *et al*, 2017).

**Figure 1:**
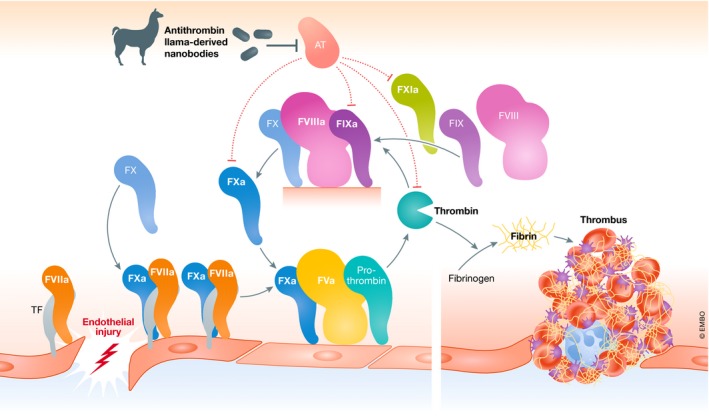
Targeted inhibition of antithrombin anticoagulant effect by nanobodies Endothelial injury results in tissue factor (TF) exposure which triggers *in vivo* hemostasis. TF first binds to FVIIa. The resultant TF:FVIIa complex is then able to active FX to FXa. In combination with its cofactor FVa, FXa then converts prothrombin into thrombin. Subsequently, thrombin back activates a series of other procoagulant factors (including FXI to FXIa, FVIII to FVIIIa, and FV to FVa). This positive feedback loop ultimately results in generation of large amounts of thrombin which convert soluble fibrinogen into insoluble fibrin that stabilizes the platelet plug. In normal plasma, this coagulation cascade is tightly regulated by the endogenous serpin antithrombin (AT) which inhibits a number of activated clotting factors including thrombin and FXa. In hemophilia, inherited deficiencies of procoagulant FVIII or FIX result in attenuated thrombin generation and consequently impaired clot stability. In this paper, Barbon *et al* demonstrate that competitive inhibition of AT activity using llama‐derived nanobodies can be used to restore thrombin generation in hemophilic plasma.

In the current paper, Barbon *et al* build upon these previous data but instead use novel engineered biparatropic sdAbs to reduce plasma AT levels and restore thrombin generation in hemophilic plasma. Unlike fitusiran siRNA which suppresses AT mRNA production within hepatocytes, these novel nanobodies function as competitive inhibitors of AT activity in plasma. There are a number of important differences between the two alternate approaches to AT inhibition. For example, following initiation of fitusiran treatment it generally requires 2–4 weeks for plasma AT levels to reach desired levels, whereas plasma AT activity levels are rapidly inhibited following nanobody infusion. Similarly, because of its mode of action, normalization of plasma AT antigen levels following discontinuation of fitusiran therapy is relatively slow (approximately 10–15% per month). In contrast, the plasma half‐life of the nanobodies is short. Moreover, since the nanobodies function as AT inhibitors, their effect can be rapidly reversed by infusion of AT concentrate. Further studies will be required to define the clinical importance of these differing approaches to target AT inhibition in PWH. Nevertheless, these exciting data undoubtedly provide us with another potential therapeutic strategy that can be utilized to develop personalized treatment regimens for PWH in the coming years.
